# Kaempferol Blocks the Skin Fibroblastic Interleukin 1β Expression and Cytotoxicity Induced by 12-O-tetradecanoylphorbol-13-acetate by Suppressing c-Jun N-terminal Kinase

**DOI:** 10.3390/nu13093079

**Published:** 2021-09-01

**Authors:** Su-Ji Park, Do-Wan Kim, Seong-Ryeong Lim, Junghee Sung, Tae Hoon Kim, In Sun Min, Chang-Hyung Choi, Sei-Jung Lee

**Affiliations:** 1 Department of Pharmaceutical Engineering, Daegu Haany University, Gyeongsan 38610, Korea; bloodslain@dhu.ac.kr (S.-J.P.); sosr200211@dhu.ac.kr (D.-W.K.); mang9811@dhu.ac.kr (S.-R.L.); 2 Research Center, Reanzen Co., Ltd., Anyang 14056, Korea; jhsung@reanzen.com; 3 FoodyWorm Inc., Yancheongsongdae-gil 10, Ochang-eup, Cheongwon-gu, Choenju-si 28118, Korea; ceo@foodyworm.com; 4 Fragrance of the Moon, 23 Taepyeong-ro, Jung-gu, Daegu 41900, Korea; etuire0209@gmail.com; 5 Division of Cosmetic Science and Technology, Daegu Haany University, Gyeongsan 38610, Korea; cchoi@dhu.ac.kr

**Keywords:** 12-O-tetradecanoylphorbol 13-acetate, apoptosis, kaempferol, normal human dermal fibroblast, reactive oxygen species

## Abstract

Kaempferol, a bioflavonoid present in fruits and vegetables, has a variety of antioxidant and anti-inflammatory capacities, but the functional role of kaempferol in oxidative skin dermal damage has yet to be well studied. In this study, we examine the role of kaempferol during the inflammation and cell death caused by 12-O-tetradecanoylphorbol-13-acetate (TPA) in normal human dermal fibroblasts (NHDF). TPA (5 μM) significantly induced cytotoxicity of NHDF, where a robust increase in the interleukin (IL)-1β mRNA among the various pro-inflammatory cytokines. The skin fibroblastic cytotoxicity and IL-1β expression induced by TPA were significantly ameliorated by a treatment with 100 nM of kaempferol. Kaempferol blocked the production of the intracellular reactive oxygen species (ROS) responsible for the phosphorylation of c-Jun N-terminal kinase (JNK) induced by TPA. Interestingly, we found that kaempferol inhibited the phosphorylation of nuclear factor-kappa B (NF-κB) and the inhibitor NF-κB (IκBα), which are necessary for the expression of cleaved caspase-3 and the IL-1β secretion in TPA-treated NHDF. These results suggest that kaempferol is a functional agent that blocks the signaling cascade of the skin fibroblastic inflammatory response and cytotoxicity triggered by TPA.

## 1. Introduction

Human skin has the two layers consisting of epidermis and the dermis, where dermal fibroblasts play an important role in the secretion of inflammatory mediators against chemical and microbial agents, maintaining the structural and mechanical properties of fibers by producing a dense extracellular matrix protein [1,2]. 12-O-tetradecanoyl phorbol-13-acetate (TPA), known as a phorbol ester, is a potent inflammagen that elicits skin edema and the cellular response of inflammation in response to the infiltration of neutrophils [3]. Increasing evidence has suggested that TPA is a tumor promoter that potentiates epidermal hyperplasia by producing reactive oxygen species (ROS) to create the oxidative stress responsible for the many human diseases [3,4]. Indeed, the ROS caused by oxidative stress has detrimental effects on the skin, such as membrane lipid peroxidation, pro-inflammatory responses, and alternation of protein and DNA structures, eventually causing skin dermal damage, necrosis, or apoptosis and leading to melanin spots, wrinkles, and age-related skin pathological process [5,6]. However, the mechanism of oxidative stress by which TPA induces the skin fibroblastic inflammation and cytotoxicity in the promoting of dermal damage had not yet been explored.

Apoptosis, characterized by morphological characteristics such as nuclear DNA fragmentation, chromatin condensation, and reduced cell volumes, is a unique cellular mechanism of programmed cell death caused by the external/internal environmental factors such as phorbol esters, oxidative stress, and mitochondrial dysfunction [7]. Given that the accumulation of oxidative molecular damage due to ROS generation in dermal fibroblasts evokes the activation of apoptotic signaling proteins and causes the abnormal expression of proinflammatory cytokines associated with various skin aging manifestations [6,8], many previous reports have suggested that dietary antioxidant intake such as phenolic compounds, flavonoids, and α-tocopherol ameliorates the risk of many types of inflammatory skin diseases [9,10].

Kaempferol, a yellow aglycone flavonoid found in broccoli, tea, strawberries, and apples, has been suggested to invoke several different mechanisms linked to the regulation of a number of key cellular signaling proteins associated with neuroprotective, anti-inflammatory, antioxidant and antimicrobial activities [11]. Accumulating evidence has reported that kaempferol possesses pharmacological effects by reducing the risk of chronic diseases, especially tumor, with much less toxic effects on the normal cells compared to standard chemotherapeutic drugs [12]. Although kaempferol has been took more attraction due to its various pharmacological functions, it is hard to deliver to organs effectively as a result of the chemical instability, hydrophobic lipophilic characters, poor water solubility [13], suggesting that the unique structure of kaempferol is one of the critical factors limiting its intraperitoneal and oral bioavailability to develop as the therapeutic, medicinal and food agents. Thus, it seems reasonable that the topical application of kaempferol as a cosmetic source to the skin would prove much more effective in ameliorating the oxidative skin dermal damage. While kaempferol has been posited to an inverse relationship with cancer induced by tumor promoters and has been found to be attractive as a treatment for many diseases given its therapeutic efficacy, its role in inflammatory skin pathogenesis during oxidative dermal damage as induced by TPA remains unclear.

In the present study, therefore, we examine the functional role of kaempferol during the inflammation and apoptosis elicited by TPA in human dermal fibroblasts and the mechanism underlying the potential pharmacological effect of kaempferol with regard to the development of therapeutic/cosmetic agent blocking the wide range of oxidative skin pathological symptoms.

## 2. Materials and Methods

### 2.1. Materials

RPMI-1640, fetal bovine serum (FBS), trypsin, 12-O-tetradecanoylphorbol-13-acetate (TPA), penicillin G, and streptomycin were purchased from Fisher Scientific (Waltham, MA, USA). Cleaved caspase-3, p-IκBα, p-p38, p-NF-κBp65, p-ERK, p-JNK, ERK, JNK, p38, IκBα, NF-κBp65, Bax and β-actin antibodies were obtained from Santa Cruz Biotechnology (Paso Robles, CA, USA). Anti-rabbit/mouse IgG secondary antibodies were obtained from Abcam (Cam-bridge, MA, USA). N-acetylcysteine (NAC) was obtained from Tocris (Minneapolis, MN, USA). Bay 11-7082, and SP600125 were purchased from Med Chem Expresss (Monmouth Junction, NJ, USA). Kaempferol was purchased from Sigma-Aldrich (St. Louis, MO, USA) and had a purity of over 98%.

### 2.2. Cells

Normal human dermal fibroblasts (NHDF), obtained from the American Type Culture Collection (ATCC, Manassas, VA, USA), were grown in RPMI-1640 containing 10% fetal bovine serum, 100 U/mL penicillin, and 100 μg/mL streptomycin, and were cultured in a dish in a 37 °C incubator with humidified atmosphere of 5% CO_2_.

### 2.3. Cell Viability Measurement

Cells were co-treated with 5 μM of TPA and kaempferol (100 nM) for 24 h. Cell viability of NHDF was evaluated by EZ-CYTOX kit (Dail-Lab Service, Seoul, Korea) as previously demonstrated [14], according to the manufacturer’s instructions. NHDF were incubated with the EZ-CYTOX master mix (10 μL) for 2 h. Cell viability was determined by using a microplate reader (SPARK, Seestrasse, Männedorf, Switzerland) at 450 nm.

### 2.4. Detection of Intracellular Reactive Oxygen Species (ROS)

NHDF was co-treated with TPA (5 μM) and kaempferol (100 nM) for various times. After that, cells were treated with 10 mM of CM-H_2_DCFDA for 30 min to quantify the level of intracellular ROS. After four washes with PBS, cells were harvested and loaded into a black 96-well plate. The fluorescence, which corresponds to the amount of intracellular ROS, was determined using a microplate reader designed for the detection of fluorescent and luminescent signals (SPARK, Seestrasse, Männedorf, Switzerland) with excitation/emission at 485/535 nm. The fluorescence signals for ROS were detected using an Olympus FluoView™ 300 confocal microscope (Center Valley, PA, USA) with 400 × objective.

### 2.5. RNA Isolation and Quantitative Real-Time Polymerase Chain Reaction (qRT-PCR)

Cells were co-treated with 5 μM of TPA and kaempferol (100 nM) for 24 h. Cellular RNA of NHDF was isolated and purified using the NucleoSpin^®^ RNA kit (Macherey-Nagel, Düren, Germany). The ratio of absorbance at 260 and 280 nm (the A260/280 ratio) was used to assess the purity of RNA preparation. A ratio higher than 1.8 was considered to be of acceptable purity. Reverse transcription for the cDNA preparation was performed with total RNA by using a ReverTra Ace® qPCR RT Master Mix (cDNA kit) (TOYOBO, Osaka, Japan). The pro-inflammatory cytokines (IL-1β, TNF-α, IL-6) evoked by TPA were amplified using a LightCycler 96 system (Roche, Basel, Switzerland) with an AccuPower^®^ 2X Greenstar qPCR Master Mix (Bioneer, Daejeon, Korea). The sequences for primer pairs used are described in Supplementary Materials Table S1. The data was collected during the extension step and analyzed using the manufacturer’s software. To verify the specificity and identity of PCR products, the amplification cycles were followed by a high-resolution melting cycle from 65 °C to 99 °C at a rate of 0.1 °C/2 s. When the melting temperature (Tm) was reached, double-stranded DNA was denatured and the SYBR was released, which caused a dramatic decrease in fluorescence intensity. The rate of this change was determined by plotting the derivative of the fluorescence relative to the temperature (dF/dT) vs. temperature by data analysis software of the real-time PCR instrument. The temperature at which a peak occurred on the plot corresponded to the Tm of the DNA duplex. β-actin was used as an endogenous control.

### 2.6. Enzyme-Linked Immunosorbent Assay (ELISA)

NHDF was treated with all inhibitors for ROS (NAC, 1 μM), NF-κB (Bay 11-7082, 1 μM), and JNK (SP600125, 1 μM) for 30 min prior to the co-treatment with TPA (5 μM) and kaempferol (100 nM) for 24 h. Culture medium from the cultured NHDF was used to quantify the secretion of human interleukin (IL)-1β. The collected medium was centrifuged at 5000 × g for 15 min to remove debris at 4 °C. The supernatants were measured using the human IL-1β ELISA kit (Abcam, Cambridge, UK) following the manufacturers’ instructions.

### 2.7. Western Blot Analysis

NHDF was co-treated with TPA (5 μM) and kaempferol (100 nM) for various times. Cellular protein was lysed with RIPA lysis buffer (ATTO Corp., Tokyo, Japan) at 37 °C for 15 min. Concentrations of protein were calculated by using the BCA assay kits (Pierce, Rockford, IL, USA). Western blot analysis was used to examine the expression of cellular signaling proteins as described earlier [15]. The quantification of band intensity measured by Scion imaging software (Scion Image 4.02, Frederick, MD, USA).

### 2.8. Statistical Analysis

Data are represented as mean value ± standard errors (S.E.). Statistical significance was analyzed by one-way analysis of variance (ANOVA) in SPSS 16 software (IBM Corp, Armonk, NY, USA). *p* < 0.05 is considered significant.

## 3. Results

### 3.1. Regulatory Effect of Kaempferol on Cytotoxicity and Inflammation Triggered by TPA

**Figure 1:**
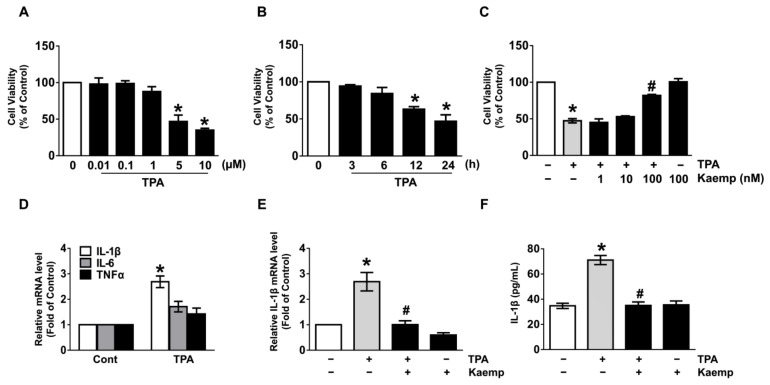
Regulatory effect of kaempferol on cytotoxicity and inflammation triggered by 12-O-tetradecanoyl phorbol-13-acetate (TPA). (**A**) Dose responses of TPA for 24 h in cell viability are shown. * *p* ≤ 0.01 vs. 0 µM. *n* = 3. (**B**) Time responses of 5 µM of TPA are shown. * *p* ≤ 0.01 vs. 0 h. *n* = 3. (**C**) Normal human dermal fibroblasts (NHDF) were treated with kaempferol and TPA for 24 h. * *p* ≤ 0.01 vs. control. # *p* ≤ 0.05 vs. TPA alone. *n* = 3. + and − represent the cells treated with and without agents, respectively. (**D**) NHDF was treated with TPA for 24 h. The effect of TPA on the expression of pro-inflammatory cytokines was evaluated by qRT-PCR. * *p* ≤ 0.001 vs. control. *n* = 3. (**E**) NHDF was exposed to the TPA in the presence of kaempferol for 24 h. The IL-1β mRNA level is shown. * *p* ≤ 0.001 versus control. # *p* ≤ 0.05 vs. TPA alone. *n* = 3. (**F**) The level of IL-1β production regulated by kaempferol in TPA-treated NHDF for 24 h was quantified by ELISA. * *p* ≤ 0.01 vs. control. # *p* ≤ 0.01 vs. TPA alone. *n* = 3.

To determine whether the 12-O-tetradecanoylphorbol-13-acetate (TPA) exerts a cytotoxic effect on human dermal fibroblasts (NHDF), we treated with TPA at concentrations of 0–10 μM for 24 h. TPA significantly induced cytotoxicity in NHDF at concentrations ranging from 5 to 10 μM compared to untreated cells (Figure 1A). An increase in cytotoxicity was showed after 24 h of treatment with 5 µM of TPA (Figure 1B). To examine the protective effect of kaempferol on the cytotoxicity of TPA, cells were treated with 5 μM of TPA and kaempferol at various concentrations 1–100 nM for 24 h. Interestingly, a treatment of 100 nM of kaempferol significantly reversed the reduced cell viability caused by TPA (Figure 1C). To determine the therapeutic potential of kaempferol, cells were treated with TPA for 1 h prior to kaempferol exposure for 24 h. We found that the cytotoxic effect of TPA is significantly blocked by the post-treatment with 100 nM of kaempferol (Supplementary Materials Figure S1A). TPA also augmented the expressions of interleukin (IL)-1β mRNA responsible for the pro-inflammatory response at 24 h, whereas for IL-6 and TNF-α, a marginal effect was noted (Figure 1D). Importantly, the increases in the expression (Figure 1E) and secretion of IL-1β (Figure 1F) induced by TPA markedly blocked by treatment with kaempferol (100 nM). These results demonstrate that the strong potential of kaempferol as a therapeutic agent on skin dermal fibroblastic cytotoxicity and inflammation triggered by TPA.

### 3.2. Kaempferol Reduces the Production of ROS in NHDF Treated with TPA

**Figure 2:**
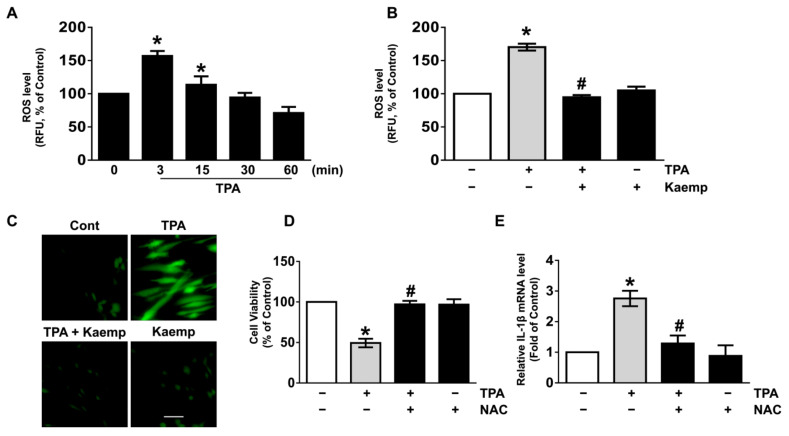
Kaempferol reduces the production of reactive oxygen species (ROS) in NHDF treated with TPA. (**A**) Time response of TPA on the level of ROS production were evaluated. * *p* ≤ 0.05 vs. 0 min. *n* = 3. RFU, relative fluorescence units. (**B**) NHDF was exposed to the kaempferol and TPA for 3 min. * *p* ≤ 0.05 vs. control. # *p* ≤ 0.01 vs. TPA alone. *n* = 3. (**C**) The blocking effect of kaempferol on ROS production induced by TPA (green) revealed by confocal microscopy is shown. Scale bars, 100 μm (magnification × 100). *n* = 3. Cells were treated with 1 μM of N-acetylcysteine (NAC) as an antioxidant for 30 min prior to TPA exposure for 24 h. The level of cell viability (**D**) and IL-1β mRNA (**E**) is shown. * *p* ≤ 0.01 versus control. # *p* ≤ 0.01 vs. TPA alone. *n* = 3.

TPA has been suggested to evoke the oxidative stress responsible for the many skin pathophysiology [3,4]. An increase in the production of reactive oxygen species (ROS) was observed at 3 min after treatment with 5 µM of TPA (Figure 2A), though the increase could be blocked by 100 nM of kaempferol (Figure 2B). The ROS scavenging effect of kaempferol was further revealed by staining NHDF with a fluorescent dye, CM-H_2_DCFDA (Figure 2C). NHDF was pre-exposed to the TPA for 3 min prior to kaempferol treatment for 3 min. Our data showed that the level of ROS evoked by TPA is markedly scavenged by the post-treatment with 100 nM of kaempferol (Supplementary Materials Figure S1B). Moreover, kaempferol (100 nM) significantly blocked the increases in ROS level on long-term exposure of TPA for 24 h in NHDF (Supplementary Materials Figure S2A). The levels of cytotoxicity (Figure 2D) and IL-1β mRNA expression (Figure 2E) induced by TPA were significantly abrogated by the incubation with an ROS inhibitor, N-acetylcysteine (NAC). These data demonstrate that the skin pharmacological effect of kaempferol on cytotoxicity and inflammation in human dermal fibroblasts is mediated by its antioxidative potential against TPA.

### 3.3. Kaempferol Uniquely Inhibits the JNK Pathways Mediated by ROS

**Figure 3:**
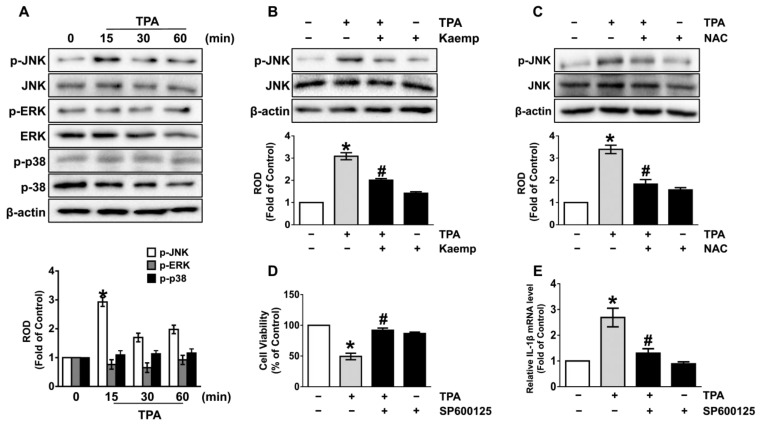
Kaempferol uniquely inhibits the c-Jun N-terminal kinase (JNK) pathways mediated by ROS. (**A**) Time response of TPA on the activation of MAPKs in NHDF were evaluated. * *p* ≤ 0.01 vs. 0 min. *n* = 3. ERK, extracellular signal-regulated kinases. ROD, relative optical density. (**B**) NHDF was exposed to the kaempferol and TPA for 15 min. * *p* ≤ 0.01 vs. control. # *p* ≤ 0.05 vs. TPA alone. *n* = 3. (**C**) Cells were treated with 1 μM of N-acetylcysteine (NAC) for 30 min prior to TPA exposure for 15 min. * *p* ≤ 0.01 vs. control. # *p* ≤ 0.01 vs. TPA alone. *n* = 3. Cells were treated with 1 μM of SP600125 for 30 min prior to TPA exposure for 24 h. The level of cell viability (**D**) and IL-1β mRNA (**E**) is shown. * *p* ≤ 0.01 vs. control. # *p* ≤ 0.01 vs. TPA alone. *n* = 3.

We next investigated how TPA is associated with the activation of mitogen-activated protein kinases (MAPKs), which are shown as downstream signaling mediators of ROS [16,17]. The activation of JNK was significantly augmented at 15 min by treatment with TPA, while the phosphorylation of ERK and p38 MAPK was not affected by the TPA in NHDF. However, the phosphorylation of JNK was attenuated by treatments with 100 nM of kaempferol (Figure 3B) and the antioxidant, NAC (Figure 3C), demonstrating that kaempferol blocks the JNK activation mediated by ROS in TPA-treated NHDF. Moreover, kaempferol (100 nM) markedly suppressed the phosphorylation of JNK on long-term exposure of TPA for 24 h in NHDF (Supplementary Materials Figure S2B). Importantly, the cytotoxicity (Figure 3D) and IL-1β expression (Figure 3E) augmented by TPA was significantly blocked by treatment with JNK inhibitor, SP600125. These data indicate that the JNK phosphorylation mediated by ROS is necessary for the dermal cell death and inflammation initiated by TPA and that the activation of the TPA signaling cascades can be blocked by kaempferol treatment.

### 3.4. The Role of Kaempferol in the Phosphorylation of NF-κB

**Figure 4:**
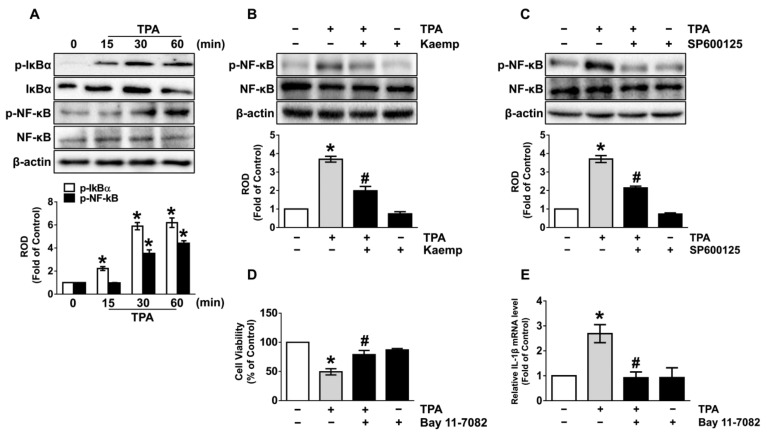
The role of kaempferol in the phosphorylation of NF-κB. (**A**) Time response of TPA on the phosphorylation IκBα and NF-κB in NHDF were evaluated. * *p* ≤ 0.01 vs. 0 min. *n* = 3. ROD, relative optical density. (**B**) NHDF was exposed to the kaempferol and TPA for 30 min. * *p* ≤ 0.01 vs. control. # *p* ≤ 0.05 vs. TPA alone. *n* = 3. (**C**) Cells were treated with 1 μM of SP600125 for 30 min prior to TPA exposure for 30 min. * *p* ≤ 0.01 vs. control. # *p* ≤ 0.01 vs. TPA alone. *n* = 3. Cells were treated with 1 μM of Bay 11-7082 for 30 min prior to TPA exposure for 24 h. The level of cell viability (**D**) and IL-1β mRNA (**E**) is shown. * *p* ≤ 0.01 vs. control. # *p* ≤ 0.05 vs. TPA alone. *n* = 3.

We then examined the role of kaempferol in the activation of transcription factor, nuclear factor-kappa B (NF-κB) mediated by the inhibitor of NF-κB (IκBα), which is required for the transcriptional expression of genes related to the inflammatory and cytotoxic responses. TPA markedly induced the phosphorylation of IκBα and NF-κB at 15 and 30 min (Figure 4A), respectively, though the increases could be attenuated by treatment with 100 nM of kaempferol (Figure 4B). Kaempferol (100 nM) also has suppressive effect on the phosphorylation of NF-κB in long-term exposure of TPA for 24 h (Supplementary Materials Figure S2C). In addition, the phosphorylation of NF-κB induced by TPA was significantly inhibited by pre-treatment with JNK inhibitor, SP600125 (Figure 4C), suggesting that NF-κB activation is downstream event of JNK activation in the dermal damage triggered by TPA. The significant increase in dermal cytotoxicity (Figure 4D) and IL-1β expression (Figure 4E) that occurred due to TPA was significantly restored after incubation with the NF-κB inhibitor, Bay 11-7082, demonstrating that kaempferol inhibits the activation of NF-κB regulated by ERK, which is required for the signaling pathway initiated by TPA during the promotion of cell death and inflammation in skin dermal fibroblast.

### 3.5. Kaempferol Blocks Dermal Fibroblastic Apoptosis and Inflammation Initiated by TPA

**Figure 5:**
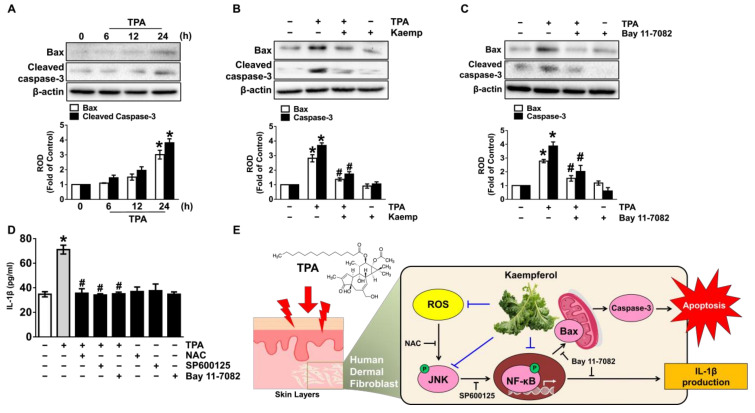
Kaempferol blocks dermal fibroblastic apoptosis and inflammation initiated by TPA. (**A**) Time response of TPA on the expression Bax and cleaved caspase-3 in NHDF were evaluated. * *p* ≤ 0.01 vs. 0 min. *n* = 3. ROD, relative optical density. (**B**) NHDF was exposed to the kaempferol and TPA for 24 h. * *p* ≤ 0.001 vs. control. # *p* ≤ 0.05 vs. TPA alone. *n* = 3. (**C**) Cells were treated with Bay11-7082 for 30 min prior to TPA exposure for 24 h. * *p* ≤ 0.001 versus control. # *p* ≤ 0.01 vs. TPA alone. *n* = 3. (**D**) Cells were treated with N-acetylcysteine (NAC), SP6001125, and Bay11-7082 for 30 min prior to TPA exposure for 24 h. The level of IL-1β production evaluated by ELISA is shown. * *p* ≤ 0.01 vs. control. # *p* ≤ 0.05 vs. TPA alone. *n* = 3. (**E**) The sequences of presumed signaling pathways regulated by kaempferol are summarized.

Given that the necessity of NF-κB in the regulation of the dermal cell death and inflammation by TPA, we further questioned how phosphorylated NF-κB actually correlates with the apoptosis-related proteins and IL-1β secretion. The expression of Bax and Cleaved Caspase-3, as representative markers of apoptosis, were evaluated in cells treated with 5 μM of TPA (Figure 5A), though the elevation could be blocked by treatment with 100 nM of kaempferol (Figure 5B). Interestingly, a pre-treatment with the Bay 11-7082 significantly blocked the expression of Bax and Cleaved Caspase-3 induced by TPA (Figure 5C), indicating that NF-κB activation elicits the transcriptional expression of apoptosis-related proteins during the skin dermal cell death triggered by TPA. These results mean that the functional effect of kaempferol on the cytotoxicity of human dermal fibroblasts is related to the blocking of apoptosis caused by TPA. Moreover, we found that the secretion of IL-1β induced by TPA significantly attenuated by treatment with the various inhibitors related to the cytotoxic signaling pathway including NAC, SP6001125, and Bay11-7082 in NHDF (Figure 5D), indicating that TPA regulates the IL-1β production involved in induction of skin dermal fibroblastic inflammation through the ROS/JNK/NF-κB pathways.

## 4. Discussion

Our findings suggest that TPA triggers skin dermal fibroblastic damage and that kaempferol neutralizes the apoptotic and inflammatory pathways induced by TPA through the inhibition of JNK-dependent IκBα/NF-κB activation occurring due to the production of ROS (Figure 5E). In general, TPA has been employed extensively as a tumor-promoting agent for the carcinogenesis of various malignant tumors in the brain [18], kidney [19] and breast [20]. Unlike the pro-tumorigenic roles, TPA shows an opposing role in the proliferation as an anti-tumorigenic agent in lymphoma, liver cancer, and other types of tumor cells via activating the different PKC isoforms and the Hippo/YAP pathway [21,22,23]. We believe that these broad and often controversial aspects of TPA are in part due to the presence of the different signaling cascade in different types of cells/tissue, and final functional results may vary depending on the cellular concept. Thus, we strongly suggest that the physiological meanings of the activation of JNK and NF-κB in the ROS signaling pathway induced by TPA in the organ carcinogenesis are different from our results revealing the TPA signaling pathway that regulates the skin dermal fibroblastic inflammation and apoptosis. Indeed, TPA can induce skin inflammation and epidermal hyperplasia with increased epidermal thickness and the infiltration of inflammatory-like cells. Moreover, TPA has been shown to evoke the ROS production and decrease the ROS detoxification enzymes in skin cells [24]. Thus, it is possible that TPA promotes apoptotic cell death signaling by producing intracellular ROS in dermal fibroblasts.

Concerning the functional role of flavonoid, it has been intensively described that flavonoid shows diverse biological properties affecting the cellular redox system to modulate signaling cascades by interacting with various enzymes [25]. Although the underlying molecular mechanisms related to the skin protective effect of kaempferol against TPA in dermal fibroblasts has not been reported, many previous studies demonstrated that kaempferol acts as a functional bioflavonoid to suppress the skin fibrosis by ameliorating the oxidative stress [26] and that it has strong antioxidant properties to fortify endogenous antioxidative defense systems and inhibit the ROS signaling pathways [27,28]. Despite the wealth of evidence indicating that kaempferol supplements can be considered safe when taken at recommended doses, the broader clinical application of kaempferol as the food agents has remained limited due to its low bioavailability following the oral intake. A previous report regarding the pharmacokinetics of kaempferol has shown that kaempferol undergoes low to moderate absorption where the oral bioavailability was found to be ~2 % of the administered doses [29]. We believe that the low bioavailability is, in part, due to the existence of its conjugated form both in plasma and urine as well as the phase I oxidative metabolism and phase II glucuronidation in the intestine, where kaempferol undergoes the oxidative conversion to quercetin followed by an isorhamnetin which could be glucuronidated [29]. Since it was proven that kaempferol is not metabolized during the cutaneous absorption [30], it seems more practical to study the topical administration of kaempferol as a cosmetic agent to the skin. Indeed, kaempferol has a high cutaneous transdermal delivery capability among the aglycone flavonoids and their corresponding glycosides in nude mice and baby pig dorsal skins. The released percentage of kaempferol that penetrated the dermal membrane appeared to be highly susceptible in stratum corneum (SC)-stripped skin and delipidated skin, suggesting intercellular lipid bilayers are a critical route for kaempferol transport [30]. These results indicate that the topical application of kaempferol as a cosmetic source to the skin would prove much more attractive as a treatment for many diseases. These data are supported by our findings revealing that kaempferol has the ability to ameliorate oxidative skin pathological symptoms including the cytotoxicity and inflammation via the scavenging of intracellular ROS induced by TPA in NHDF. Indeed, kaempferol has been reported to attenuate skin aging manifestations caused by oxidative stress, such as a loss of skin elasticity, wrinkle formation, and inflammatory responses [31]. These findings also therefore suggest that kaempferol is a potent prophylactic and therapeutic flavonoid that can suppress the skin dermal fibroblastic damages.

To gain insight into how ROS production is connected with the signaling cascades of the skin fibroblastic inflammatory response and cytotoxicity, we further determined the role of mitogen-activated protein kinases (MAPKs) with respect to their possible roles in the modulation of gene expression, proliferation, inflammation and programmed cell death as down-stream mediators of ROS [15,32]. Despite the mounting evidence of the influence of MAPK on the ROS signaling pathway, we noted that kaempferol has an inhibitory effect on the activation of JNK as uniquely phosphorylated by TPA. This result is further supported by a previous study demonstrating that ERK is a critical for the promoting of cell survival, whereas JNK and p38-MAPK were shown to be involved in various stress responses leading to the induction of apoptosis [32]. Indeed, JNK activation is a distinctive step in many inflammatory skin pathogeneses in response to stressful stimuli induced by TPA [33,34], suggesting that JNK is a critical signaling mediator of TPA during the skin fibroblastic inflammation and apoptotic cell death in dermal fibroblasts. Moreover, we found significant evidence that ROS signaling is required for JNK phosphorylation to elicit the apoptotic cell death pathways triggered by TPA. Hence, our findings demonstrate that the pharmacological effect of kaempferol on abnormal dermal JNK activation is mediated by its antioxidative potential against TPA.

Given that ROS and JNK are major effector molecules of kaempferol in TPA-treated NHDF cells, we tried to know the mechanism by which these effectors stimulate the other molecules that are important during the cytotoxic and inflammatory processes. NF-κB is a multifaceted transcriptional regulator that moves into the nucleus during the activation of IκBα to regulate gene expressions related to the apoptotic and inflammatory responses [35]. In this study, our results revealed that kaempferol has an inhibitory effect on the activation of NF-κB mediated by JNK phosphorylation to attenuate the level of cytotoxicity and IL-1β production as evoked by TPA. Considering the effect of JNK on the NF-κB activity, many previous studies demonstrated that the JNK activated by ROS production is able to stimulate the transcriptional activation of NF-κB in the promoting of inflammation and apoptosis [36]. Consistent with our results, previous work has reported that NF-κB is a central transcriptional factor of the inflammatory and apoptotic signaling pathways in dermal fibroblasts during the skin aging process [37]. Hence, it is conceivable that kaempferol harbors an important function in the blocking the NF-κB-mediated apoptotic pathway through the inhibition of oxidative JNK activation. Together, our results suggest that kaempferol is a functional agent that blocks the skin dermal fibroblastic inflammatory and apoptotic cell death coupled with ROS production responsible for the activation of the JNK and NF-κB pathways induced by TPA.

Finally, the present results demonstrate that kaempferol has the ability to restore the levels of Bax and caspase-3 via the suppression of NF-κB activity in TPA-stimulated NHDF. Earlier work suggested that the NF-κB activated by various tumor promoters influences the expressions of Bcl-2 family proteins responsible for the cell death pathway mediated by the mitochondria [38,39]. Indeed, Bax has been shown to possess a promoter region as a specific binding site for NF-κB during the apoptotic cell death mediated by the mitochondria [40]. Given that oxidative stress triggers apoptotic cell death via mitochondrial Bax oligomerization, Bax has been known as an important determining protein for apoptotic susceptibility [41]. The mitochondrial translocation of Bax induces the corresponding oligomer formation process, leading to the increase in mitochondrial membrane permeabilization responsible for the cytochrome c release and caspase-9 activation processes required for caspase-3 activation [42]. Interestingly, we have shown convincing data showing TPA regulates the IL-1β production involved in induction of skin dermal fibroblastic inflammation through the ROS/JNK/NF-κB pathways. These results are further supported by previous results showing that Gasdermin family, a key protein that mediates non-canonical pyroptosis, is able to regulate the releasing mature pro-inflammatory cytokine IL-1β mediated by the active caspase-3 in the switching between apoptosis and pyroptosis [43]. Although further research is required to establish in greater detail in the relationship between apoptosis and inflammation induced by TPA, we suggest our results are the first report demonstrating the signaling cascade of the apoptotic cell death associated with IL-1β production in skin dermal fibroblast during the TPA exposure. Thus, these results imply that kaempferol has the unique function of being able to suppress the oxidative skin dermal damage caused by TPA exposure as part of the amelioration of the activation of the inflammatory and mitochondrial apoptotic pathways.

## 5. Conclusions

Collectively, our findings highlight the relevance of the pharmacological effect of kaempferol in the blocking of a wide range of oxidative skin pathological symptoms. Moreover, we suggest the novel mechanism of kaempferol to ameliorate the ROS/JNK/NF-κB signaling pathway during the TPA exposure is likely to be critical to develop the therapeutic and cosmetic agents against the skin dermal fibroblastic inflammation and oxidative damage responsible for the most common consequences of accumulative changes in the promoting skin pathophysiology. Further research is required to establish in greater detail in the encapsulation of kaempferol with different formulations using hydrogels, micelles, and nanoparticles as a drug-delivery system that stabilizes the physical and chemical properties of kaempferol in terms of cellular uptake, biodistribution, and accumulation at sites of interest.

## Data Availability

Data is contained within the article and supplementary material.

## Supplementary Materials

The following are available online at https://www.mdpi.com/article/10.3390/nu13093079/s1, Figure S1: The therapeutic potential of kaempferol on cytotoxicity and ROS production triggered by TPA, Figure S2: The regulatory effect of kaempferol on the production of ROS and the phosphorylation of JNK and NF-κB for long-term exposure of TPA in NHDF, Table S1: PCR primer sequences.

## Abbreviations

JNK: c-Jun N-terminal kinases; TPA: 12-O-tetradecanoylphorbol-13-acetate; NF-κB: Nuclear factor-kappa B; NHDF: Normal human dermal fibroblasts; IL-1β: interleukin-1β; ROS: Reactive oxygen species; Bax: Bcl-2 associated X protein; MAPKs: Mitogen-activated protein kinases; NAC: N-acetylcysteine.
