# Supplementary material for: Kaempferol Blocks the Skin Fibroblastic Interleukin 1β Expression and Cytotoxicity Induced by 12-O-tetradecanoylphorbol-13-acetate by Suppressing c-Jun N-terminal Kinase
Source: Nutrients. 2021 Sep 1;13(9):3079. doi: 10.3390/nu13093079 (PMC8466288; doi:10.3390/nu13093079)
Supplement: Supplementary file 1 [file nutrients-13-03079-s001.zip › nutrients-1357330-supplementary.pdf]

Supplementary Materials

Kaempferol blocks the skin fibroblastic interleukin 1β expres-sion and cytotoxicity induced by 12-O-tetradecanoylphorbol-13-acetate by suppressing c-Jun N-terminal kinase

Su-Ji Park [1], Do-Wan Kim [1], Seong-Ryeong Lim [1], Junghee Sung [2], Tae Hoon Kim [3], In Sun Min [4], Chang-Hyung Choi [5] and Sei-Jung Lee [1,*]

[1] *Department of Pharmaceutical Engineering, Daegu Haany University, Gyeongsan 38610, Korea;*

[2] *Research Center, Reanzen Co. Ltd., Anyang 14056, South Korea*

[3] *FoodyWorm Inc., Yancheongsongdae-gil 10, Ochang-eup, Cheongwon-gu, Choenju-si 28118, South Korea;*

[4] *Fragrance of the Moon, 23 Taepyeong-ro, Jung-gu, Daegu 41900, Korea*

[5] *Division of Cosmetic Science and Technology, Daegu Haany University, Gyeongsan 38610, Korea*

*** Corresponding authors**: Sei-Jung Lee Ph.D.

Department of Pharmaceutical Engineering, Daegu Haany University

Gyeongsan 38610, South Korea

E-mail: sjlee@dhu.ac.kr; Tel: 82-54-819-1806; Fax: 82-54-819-1406

Table S1. PCR primer sequences

Gene	Identification	Primer sequence, 5`-3`
IL-1β	Forward	TTCGAGGCACAAGGCACAAC
	Reverse	GTGGTGGTCGGAGATTCGTA
TNF-α	Forward	CTCCTCACCCACACCATCA
	Reverse	GGAAGACCCCTCCCAGATAG
IL-6	Forward	CAATAACCACCCCTGACCCAA
	Reverse	ACCAGAAGAAGGAATGCCCA
β-actin	Forward	AACCGCGAGAAGATGACCCAGATCATGTTT
	Reverse	AGCAGCCGTGGCCATCTCTTGCTCGAAGTC

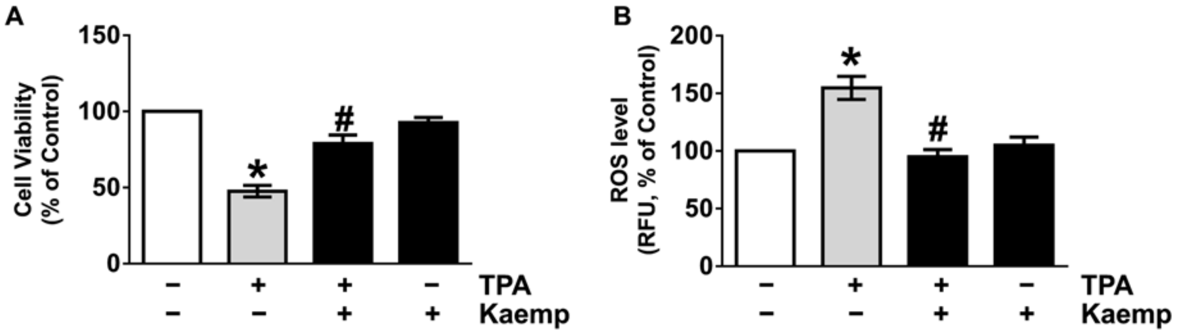

Figure S1. The therapeutic potential of kaempferol on cytotoxicty and ROS production triggered by TPA. (A) NHDF was treated with 5 μM of TPA for 1 h prior to kaempferol (100 nM) exposure for 24 h. Cell viability determined by EZ-CYTOX kit is shown. * $p \leq 0.01$ versus control. # $p \leq 0.05$ versus TPA alone. $n = 3$. (B) Cells were treated with TPA for 3 min prior to kaempferol exposure for 3 min. ROS production determined by staining NHDF with CM-H_2DCFDA is shown. * $p \leq 0.01$ versus control. # $p \leq 0.01$ versus TPA alone. $n = 3$. RFU, relative fluorescence units.

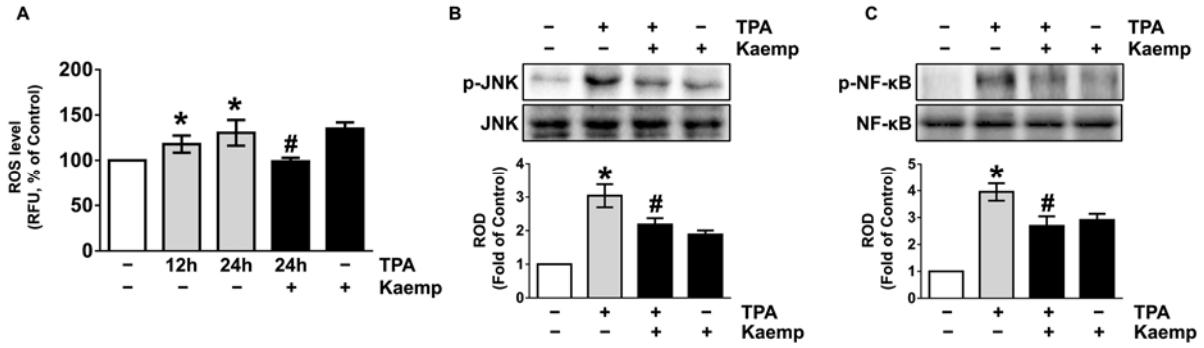

Figure S2. The regulatory effect of kaempferol on the production of ROS and the phosphorylation of JNK and NF-κB for long-term exposure of TPA in NHDF. NHDF was treated with kaempferol and TPA for 24 h. (A) ROS production determined by staining NHDF with CM-H_2DCFDA is shown. * $p \leq 0.05$ versus control. # $p \leq 0.01$ versus TPA alone. $n = 3$. RFU, relative fluorescence units. The effect of kaempferol on the phosphorylation of JNK (B) and NF-κB (C) determined by western blot is shown. * $p \leq 0.01$ vs. control. # $p \leq 0.01$ vs. TPA alone. $n = 3$. ROD, relative optical density.
